# High Prevalence of SARS-CoV-2 Omicron Infection Despite High Seroprevalence, Sweden, 2022

**DOI:** 10.3201/eid2906.221862

**Published:** 2023-06

**Authors:** Ramona Groenheit, Philip Bacchus, Ilias Galanis, Klara Sondén, Ioana Bujila, Tatiana Efimova, Fredrik Garli, Oskar Karlsson Lindsjö, Mikael Mansjö, Elin Movert, Aleksandra Pettke, Marie Rapp, Maike Sperk, Sandra Söderholm, Karin Valentin Asin, Sarah Zanetti, Maria Lind Karlberg, Andreas Bråve, Kim Blom, Jonas Klingström

**Affiliations:** Public Health Agency of Sweden, Solna, Sweden (R. Groenheit, I. Galanis, K. Sondén, I. Bujila, T. Efimova, F. Garli, O. Karlsson Lindsjö, M. Mansjö, E. Movert, A. Pettke, M. Rapp, M. Sperk, S. Söderholm, K. Valentin Asin, S. Zanetti, M. Lind Karlberg, A. Bråve, K. Blom, J. Klingström);; Swedish Armed Forces, Umeå, Sweden and Lund University, Lund, Sweden (P. Bacchus);; Karolinska Institutet, Stockholm, Sweden (K. Sondén, M. Sperk, K. Blom, J. Klingström);; Linköping University, Linköping, Sweden (J. Klingström)

**Keywords:** COVID-19, respiratory infections, SARS-CoV-2, SARS, coronavirus disease, zoonoses, viruses, coronavirus, point prevalence, epidemiology, vaccines, seroprevalence, symptoms, PCR, Omicron, Sweden

## Abstract

We performed 2 surveys during 2022 to estimate point prevalences of SARS-CoV-2 infection compared with overall seroprevalence in Sweden. Point prevalence was 1.4% in March and 1.5% in September. Estimated seroprevalence was >80%, including among unvaccinated children. Continued SARS-CoV-2 surveillance is necessary for detecting emerging, possibly more pathogenic variants.

The SARS-CoV-2 Omicron variant has had strong effects on the COVID-19 pandemic. New Omicron subvariants have emerged over time and those subvariants have increased capacity to evade neutralizing antibody responses induced by both vaccines and prior infections, causing breakthrough infections and reinfections (*1*–*4*). After general PCR testing was halted in Sweden in early 2022, the possibility to track the COVID-19 situation and maintain surveillance in the general population largely depended on point prevalence surveys to detect acute SARS-CoV-2 infection by using PCR and estimates of previous infections by using serology. We performed 2 cross-sectional surveys during 2022 to estimate SARS-CoV-2 point prevalences and overall seroprevalence in Sweden. 

## The Study

Participants were invited from a nationwide probability-based web panel (*5*,*6*). Participants received material for sampling and instructions on how to perform self-sampling at home (Appendix). The first survey, March 21–25, covered all 21 regions in Sweden; the second survey, September 26–29, covered 11 regions in the country, representing 64% of the population. We performed surveys as part of the Public Health Agency of Sweden’s assignment to monitor communicable diseases and evaluate infection control measures (in accordance with §§18 of Ordinance 2021:248 from the Swedish Parliament). All participants provided informed consent, and the legal guardian provided consent for persons <16 years of age.

In March, 11,334 persons were invited and 2,906 persons 2–96 years of age participated (Appendix Table 1). In total, we analyzed 2,659 samples for ongoing infection and 2,587 samples for serologic responses (Figure, panel A). We detected 48 PCR-positive samples showing SARS-CoV-2 infection, an estimated point prevalence of 1.4% (95% CI 0.9%–2.1%) in the population of Sweden at the end of March. One infection was caused by the Delta variant and 47 by Omicron subvariants (Appendix Table 2). Data from the national registry for communicable diseases revealed that 633 (24%) of the 2,659 participants with a valid PCR result had >1 previous confirmed SARS-CoV-2 infection; 79.4% had received >3 vaccine doses (Table 1). Among 48 participants with PCR-positive results, 4 (8.3%) had previously reported SARS-CoV-2 infections. On the basis of spike IgG data from the 2,587 participants with a valid sample for serology, we estimated a 93.3% (95% CI 91.5%–94.8%) SARS-CoV-2 seroprevalence in the population of Sweden at the end of March 2022. The estimated seroprevalence was 80.1% (95% CI 71.1%–87.4%) in children <11 years of age and 94.2%–98.8% in persons >11 years of age (Table 2).

**Figure:**
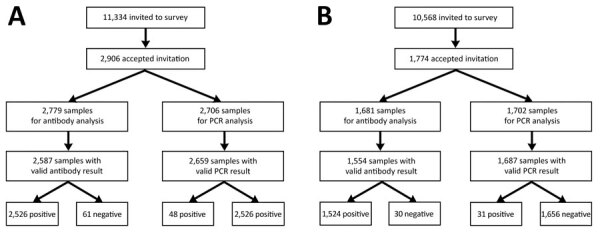
Flowchart of study participant enrollment and collected and analyzed samples in a study of prevalence of SARS-CoV-2 Omicron infection despite high seroprevalence, Sweden, 2022. A) Surveys performed during March 21–25. B) Surveys performed September 26–29. Point prevalence and Omicron subvariant data from the September study was published previously (*6*).

**Table 1:** Number of COVID-19 vaccine doses received by participants in surveys conducted for study of high prevalence of SARS-CoV-2 Omicron infection despite high seroprevalence, Sweden, 2022

No. doses	No. participants (%)	
	March 21–25, n = 2,659	September 26–29, n = 1,687
0	178 (6.9)	96 (6.2)
1	12 (0.5)	5 (0.3)
2	343 (13.3)	126 (8.1)
3	2,006 (77.5)	809 (52.1)
4	48 (1.9)	408 (26.3)
5	0	110 (7.1)

**Table 2:** Estimated SARS-CoV-2 seroprevalence by age group in study of high prevalence of SARS-CoV-2 Omicron infection despite high seroprevalence, Sweden, 2022

Age group	% Participants (95% CI)	
	March 21–25, n = 2,587	September 26–29, n = 1,554
1–11	80.1 (71.1–87.4)	84.0 (70.3–93.3)
12–19	97.2 (81.9–100.0)	84.9 (53.2–99.0)
20–29	95.9 (89.6–99.2)	92.7 (70.8–100)
30–49	94.2 (89.4–97.5)	95.9 (89.7–99.0)
50–64	95.3 (91.6–97.9)	95.0 (88.3–98.7)
65–79	95.0 (94.2–95.7)	96.7 (89.2–100.0)
>80	98.8 (89.8–100.0)	100 (94.3–100.0)

In the September survey, 10,568 persons were invited and 1,774 persons 2–94 years of age participated. We were able to analyze 1,687 samples for ongoing infection (Appendix Table 1), and 1,554 samples for serologic responses (Figure, panel B). We previously reported that 31 of 1,687 participants were PCR-positive in September, that the estimated point prevalence was 1.5% (95% CI 0.9%–2.5%), and all infections were caused by Omicron subvariants (*6*). Among participants in the September survey, 485 (29%) had >1 previously confirmed SARS-CoV-2 infection; 85.5% had received >3 vaccine doses (Table 1). Among PCR-positive participants, 22 (71%) had previously reported SARS-CoV-2 infections. On the basis of spike IgG data (n = 1,554), we estimated that SARS-CoV-2 seroprevalence in Sweden was 93.1% (95% CI 89.2%–96.0%) at end of September. Estimated seroprevalence was 84.0% (95% CI 70.3%–93.3%) in persons 2–11 years of age and 84.9%–100% in persons >11 years of age (Table 2).

Using answers in the participant symptom survey, we next analyzed for symptoms in general and for symptoms among SARS-CoV-2–infected participants. Overall, 65.2% of participants in March and 67.7% of participants in September, experienced >1 symptom within 2 weeks before sampling. Of participants with negative PCR results, 64.6% in March and 67.3% in September had >1 symptom (Appendix Table 3). Among 79 PCR-positive participants, 4 had no symptoms within 2 weeks before sampling (Appendix Table 3), and 3 of those 4 experienced symptoms within 1 week after sampling.

## Conclusions

Beginning February 9, 2022, mainly hospitalized persons, healthcare workers, long-term care facility staff, and at-risk persons with symptoms indicating COVID-19, were tested for SARS-CoV-2 infection in Sweden. Because the general population was no longer tested, trends in prevalence and transmission patterns were difficult to assess in real-time. To estimate point prevalence of infection in the population, Sweden needed random sampling of the population on a nationwide level.

We estimated point prevalences in Sweden of 1.4% during March 21–25 and, as previously reported (*6*), 1.5% during September 26–29. Those estimated point prevalences were higher than those in our previous national surveys (*5*). In another survey of healthcare workers in Stockholm during June 28–29, 2022, we observed asymptomatic SARS-CoV-2 infections in 2.3% of participants (*7*). Although healthcare workers are likely at higher risk for infection than the general population, those 3 surveys collectively indicated a continued high level of Omicron transmission in Sweden during March, June, and September 2022. Similar, or even higher, point prevalences were reported from other countries during 2022. For example, surveys performed on the general population in the United Kingdom estimated point prevalences of 6.7%–8.7% in March and 2.1%–2.5% in September for England, Scotland, Wales, and Northern Ireland (*8*). Moreover, a survey of blood donors in Denmark estimated that, by March 2022, ≈66% of the age-matched healthy population had been infected by SARS-CoV-2 in <5 months (*9*). Taken together, those and many other reports show the high capacity of Omicron to spread, including among highly vaccinated populations.

A large percentage of the population were positive for spike IgG in March and in September 2022, which can partly be explained by the high vaccine coverage in Sweden. COVID-19 vaccination was not recommended for children <12 years of age in Sweden. Hence, in the youngest, unvaccinated, age group, seroconversion was likely induced solely by infection, indicating that a large percentage (80%) of that age group had already been infected with SARS-CoV-2 by March 2022. Similar levels of infections in children have been reported from Bavaria, Germany, including seroprevalences of 67% for preschool children 1–4 years of age and 84% for school-age children 5–10 years of age in June 2022, largely caused by the Omicron variant (*10*).

Current vaccines seem to provide only limited, short-term, inhibitory effect on Omicron transmissibility (*11*). Of note, our surveys showed that among PCR-positive participants in March, 8.3% had a previously recorded SARS-CoV-2 infection, but in September, those participants increased to 71%, indicating a high level of reinfection caused by then circulating Omicron subvariants, which have shown highly increased capacity to avoid neutralizing antibodies (*12*–*14*).

In summary, we estimate that ≈1 of every 66 persons in Sweden was infected with SARS-CoV-2 by March and September 2022. Although Omicron has a high transmission capacity, current vaccines protect against severe disease, as noted by the low fatality rate observed in Omicron-infected persons in Denmark (*9*). However, because Omicron has the capacity to efficiently transmit despite high vaccine coverage, continued surveillance of the general population for early signs of new, more possibly pathogenic, emerging SARS-COV-2 variants remains crucial.

Appendix Additional information on high prevalence of SARS-CoV-2 Omicron infection despite high seroprevalence, Sweden, 2022.
