# Supplementary material for: High Prevalence of SARS-CoV-2 Omicron Infection Despite High Seroprevalence, Sweden, 2022
Source: Emerg Infect Dis. 2023 Jun;29(6):1240–3. doi: 10.3201/eid2906.221862 (PMC10202879; doi:10.3201/eid2906.221862)
Supplement: Appendix — Additional information on high prevalence of SARS-CoV-2 Omicron infection despite high seroprevalence, Sweden, 2022. [file 22-1862-Techapp-s1.pdf]

Article DOI: https://doi.org/10.3201/eid2906.221862

EID cannot ensure accessibility for supplementary materials supplied by authors. Readers who have difficulty accessing supplementary content should contact the authors for assistance.

High Prevalence of SARS-CoV-2 Omicron Infection Despite High Seroprevalence, Sweden, 2022

Appendix

Methods

Survey Population

This investigation was an observational, cross-sectional study. Individuals were invited to participate based on a nationwide probability-based web panel regularly used for health-related questionnaires at the Public Health Agency of Sweden, including previous point-prevalence studies (*1*,*2*). All participants provided informed consent. For those under 16 years of age, the legal guardian was asked to, and provided, informed consent. Participation could be withdrawn at any time.

The surveys were performed as part of the Public Health Agency of Sweden’s assignment to monitor communicable diseases and evaluate infection control measures in accordance with §§18 of the ordinance (2021:248) from the Swedish Parliament.

Self-Sampling

Participants received material for sampling and instructions on how to perform self-sampling at home. Self-sampling kits for PCR-test contained a sterile cotton swab and a test tube containing 0.5 ml 0.9% saline. Participants performed self-sampling using a cotton swab for swabbing of the pharynx followed by swabbing of the outer nostrils using the same swab. Participants then drooled/spat into a cup and swirled the same cotton swab in the saliva before finally moving the swab to the test tube for 30 seconds of swirling. The swab was then removed, and the test tube tightly closed.

Self-sampling kit for blood sampling contained a device for finger picking and paper for collection of blood on a qDBS (Capitainer). The participants were asked to store the samples in a refrigerator until collection for transport to laboratories for further analysis.

PCR and Sequencing

Samples were analyzed for SARS-CoV-2 by using real-time reverse transcription PCR (RT–PCR) assays routinely used to diagnose COVID-19 in Sweden at the National Pandemic Center, Stockholm, Sweden. To verify that a sample was taken correctly in terms of swabbing against mucosal surfaces in the nose or throat, RNase P mRNA was analyzed. Samples negative for RNase P were excluded from analysis. RT-PCR–positive samples were subsequently sequenced at the Public Health Agency of Sweden, Solna, and SARS-CoV-2 variants were identified based on Pangolin version 4.1.3 and pangolin-data version 1.15.1 (*3*,*4*).

Serologic Response

Samples were extracted from the qDBS, as previously described (*5*,*6*), and then analyzed for spike IgG by using an ELISA with a stringent cutoff for positive detection of S-specific IgG set to optical density (OD) 0.7 or higher, as previously described (J.W. Byström et al., unpub. data).

Statistical Analyses

We estimated the proportion of SARS-CoV-2–positive individuals and the proportion with antibodies to SARS-CoV-2 in the Swedish population and in 11 regions in the second study as a weighted proportion. The weights were based on the sampling weights of the participants in the web panel, calibrated with the GREG estimator (*7*) to adjust for nonresponse bias. The auxiliary information used in the calibration consisted of age, gender, geographic region, and vaccination coverage with at least 2 doses by age group, up to 2 weeks before the first sampling date. The seroprevalence estimates were adjusted to take into account the sensitivity (99.2%) and specificity (99.3%) of the test with the Rogan-Gladen formula (*8*). All estimates are reported with their respective 95% CI that were calculated using the modified Clopper-Pearson method (*9*). Analyses were performed in R (https://www.r-project.org) using the “survey” package version 4.1-1.

References

1. Public Health Agency of Sweden. Health report – a web panel [in Swedish] [cited 2023 Feb 8]. https://www.folkhalsomyndigheten.se/folkhalsorapportering-statistik/om-vara-datainsamlingar/halsorapport/

2. Groenheit R, Beser J, Kühlmann Berenzon S, Galanis I, van Straten E, Duracz J, et al. Point prevalence of SARS-CoV-2 infection in Sweden at six time points during 2020. BMC Infect Dis. 2022;22:861. PubMed https://doi.org/10.1186/s12879-022-07858-6

3. O'Toole Á. Pangolin v4.1.3 [cited 2022 Oct 20]. https://github.com/cov-lineages/pangolin/releases/tag/v4.1.3

4. Hinrichs A. Pangolin-data v1.15.1 [cited 2022 Oct 20]. https://github.com/cov-lineages/pangolin-data/releases/tag/v1.15.1

5. Byström JW, Vikström L, Rosendal E, Gröning R, Gwon YD, Nilsson E, et al. At-home sampling to meet geographical challenges for serological assessment of SARS-CoV-2 exposure in a rural region of northern Sweden, March to May 2021: a retrospective cohort study. Euro Surveill. 2023;28:2200432. PubMed https://doi.org/10.2807/1560-7917.ES.2023.28.13.2200432

6. Normark J, Vikström L, Gwon YD, Persson IL, Edin A, Björsell T, et al. Heterologous ChAdOx1 nCoV-19 and mRNA-1273 vaccination. N Engl J Med. 2021;385:1049–51. PubMed https://doi.org/10.1056/NEJMc2110716

7. Särndal C-E, Swensson B, Wretman J. Model assisted survey sampling. New York: Springer; 1991.

8. Rogan WJ, Gladen B. Estimating prevalence from the results of a screening test. Am J Epidemiol. 1978;107:71–6. PubMed https://doi.org/10.1093/oxfordjournals.aje.a112510

9. Korn E, Graubard B. Confidence intervals for proportions with small expected number of positive counts estimated from survey data. Survey Methodology. 1998;24;193–201.

Appendix Table 1. Age and sex distribution of participants in the surveys with a positive or negative SARS-CoV-2 PCR test, Sweden 2022*

	March, no.			September, no.		
Age group, y	F	M	Total no. (%)	F	M	Total no. (%)
1–15	96	77	173 (6.5)	53	40	93 (5.5)
16–29	140	52	192 (7.2)	82	20	102 (6.0)
30–59	885	426	1,311 (49.3)	568	260	828 (49.1)
≥60	523	460	983 (37.0)	360	304	664 (39.4)
Total no. (%)	1,644 (61.8)	1,015 (38.2)	2,659 (100)	1,063 (63.0)	624 (37.0)	1,687 (100)

*Surveys conducted during March 21–25 and September 26–29.

Appendix Table 2. Identified SARS-CoV-2 variants among the PCR-positive samples in March (N = 48).

SARS-CoV-2 variant	No. (%)
AY.111	1 (2.1)
BA.1	1 (2.1)
BA.2	33 (68.8)
BA.2.9	13 (27.1)

Appendix Table 3. Distribution of symptoms within 2 weeks before sampling among participants with a positive or negative SARS-CoV-2 PCR-test, Sweden, 2022.

	March		September	
Symptom	% Infected, n = 48	% Not infected, n = 2,602	% Infected, n = 31	% Not infected, n = 1,648
Runny nose	81.3	28.1	67.7	28.3
Cough	75	16.3	71.0	21.2
Headache	66.7	32.5	71.0	36.8
Sore throat	66.7	14.6	58.1	20.3
Huskiness	56.3	8.0	38.7	10.7
Extreme fatigue, exhaustion	54.2	18.6	38.7	22.6
Fever	47.9	5.7	41.9	7.0
Myalgia	35.4	13.2	32.3	15.5
Chills	25	4.1	25.8	5.0
Joint pain	25	12.5	25.8	14.4
Stomach ache	25	14.2	12.9	13.8
Shortness of breath, difficulty breathing	22.9	6.5	12.9	8.7
Ear pain	20.8	4.1	22.6	4.3
Diarrhea	20.8	9.7	19.4	10.1
Eye discharge	18.8	5.3	16.1	5.6
Nausea	16.7	8.7	12.9	9.5
Loss of smell	10.4	3.4	9.7	3.5
Skin rashes†	8.3	4.5	6.5	4.6
Chest pain	6.3	2.7	6.5	2.7
Loss of taste	6.3	2.6	6.5	2.8
Nose bleeds	4.2	5.7	3.2	3.8
Vomiting	0	2.2	9.7	0.9
No symptoms	2.1	35.4	9.7	32.7

*Surveys conducted during March 21–25 (n = 2,650) and September 26–29 (n = 1,679).
†Includes hives, dots, pustules, or blisters.
